# The ant genus *Tetramorium* Mayr in the Afrotropical region (Hymenoptera, Formicidae, Myrmicinae): synonymisation of *Decamorium* Forel under *Tetramorium*, and taxonomic revision of the *T. decem* species group

**DOI:** 10.3897/zookeys.411.7260

**Published:** 2014-05-28

**Authors:** Francisco Hita Garcia, Brian L. Fisher

**Affiliations:** 1 Entomology Department, California Academy of Sciences, 55 Music Concourse Drive, San Francisco, CA 94118, U.S.A.

**Keywords:** Afrotropical region, *Decamorium*, taxonomy, Tetramoriini, *Tetramorium*, *T. decem* species

## Abstract

In this study we synonymise the genus *Decamorium* Forel under *Tetramorium* Mayr, revise the new *T. decem* species group by providing a diagnosis of the group, an illustrated identification key to species level, and worker-based species descriptions for all five species, which include diagnoses, discussions, images, and distribution maps. The following species are revised in this study: *T. decem* Forel, **comb. r.**, *T. raptor* **sp. n.**, *T. uelense* Santschi, **comb. r.**, *T. ultor* Forel, **comb. r.**, **stat. r. & stat. n.**, and *T. venator* **sp. n.** In addition, we also designate lectotypes for *T. decem*, *T. uelense*, and *T. ultor*.

## Introduction

The genus *Tetramorium* Mayr is globally distributed and with 520 valid species it represents one of the most species-rich ant genera (Bolton 2014). The vast majority of these are found in the tropics of the Old World. In the Afrotropical and Malagasy regions, *Tetramorium* is hyperdiverse by the definitions of Wilson (2003) and Moreau (2008). In Madagascar and the neighbouring islands of the Indian Ocean, recent studies have revealed a highly endemic and astonishingly diverse *Tetramorium* fauna consisting of around 120 species (Bolton 1979; Hita Garcia and Fisher 2011, 2012a, 2012b, unpublished). The known Afrotropical *Tetramorium* fauna was thoroughly revised by Bolton (1976, 1980, 1985), parts of which were recently updated by Hita Garcia et al. (2010) and Hita Garcia and Fisher (2011, 2013), producing a current total of 224 species. In addition, there are at least 100 more undescribed Afrotropical species located in several museum collections awaiting formal description (FHG, unpublished data). Traditionally, what is now considered as *Tetramorium* was divided into the genera *Atopula* Emery, *Macromischoides* Wheeler, *Tetramorium*, and *Xiphomyrmex* Forel until Bolton’s genus-level revision (1976). The name *Tetramorium* was used for a much smaller subset of species with twelve antennal segments. Bolton provided ample evidence for the artificiality of these genera and synonymised them under *Tetramorium*. Particularly noteworthy is the fact that the previous separation of *Xiphomyrmex* (11-segmented antennae) from *Tetramorium* (12-segmented antennae) was based solely on the difference in the antennomere count; Bolton showed this character to be variable in other tetramoriine genera.

Forel (1913a) described *Decamorium* Forel as a subgenus of *Tetramorium* on the basis of the ten-segmented antennae and the very pronounced and deep antennal scrobes. A few years later Arnold (1917) followed Forel and also treated *Decamorium* as a subgenus of *Tetramorium*. He based his decision on the ten-segmented antennae, the well-defined and deep antennal scrobes, the obsolete lateral ridges of the clypeus, and the strongly swollen tibiae and femorae in the worker caste. Nevertheless, apart from these two works (Forel 1913a; Arnold 1917), most other authors (and later even Forel himself) have treated *Decamorium* as a genus distinct from *Tetramorium* (Emery 1914, 1924; Forel 1917; Wheeler 1922; Bernard 1953; Bolton 1973, 1976, 1995). In his classification of the Myrmicinae, Emery (1914) was the first to treat *Decamorium* as a “genus” rather than a subgenus, although he did not provide any explanation of his decision. Later, in his “Genera Insectorum”, Emery (1924) continued to list *Decamorium* as a genus. In that work he re-described the genus and separated it from the other then known tetramoriine genera, again on the basis of the ten-segmented antennae of the workers and queens. All subsequent authors listed *Decamorium* as a genus without taxonomic treatment until Bolton’s (1976) revision of the tribe Tetramoriini. As mentioned above, he diagnosed most of the currently valid genera of the tribe and also reviewed *Decamorium*. Bolton (1976) clearly stated that the separation of *Decamorium* from *Tetramorium* on the grounds of the reduced antennal count, reduced clypeal shield, and differences in mandibular dentition was relatively dubious. He doubted that these characters would persist to diagnose *Decamorium* in the future. However, since then nothing more on the generic limits or alpha taxonomy of *Decamorium* has been published, and all authors continued to list *Decamorium* as a distinct genus (e.g. Hölldobler and Wilson 1990; Bolton 1995, 2003, 2014; Robertson 2000; Hita Garcia et al. 2013).

In this study we propose *Decamorium* as a junior synonym of *Tetramorium* and lower it to the arbitrary rank of a species group. Our decision is based on a critical analysis of the diagnostic characters previously defining *Decamorium*. In addition, we revise the alpha taxonomy of the *Tetramorium decem* species group. A diagnosis of the *Tetramorium decem* species group is given together with an illustrated identification key to species on the basis of the worker caste. In addition, all members of the species group are described/re-described including diagnoses, discussions, high-quality montage images and distribution maps.

### Abbreviations of depositories

The collection abbreviations follow Evenhuis (2014). The material upon which this study is based is located and/or was examined at the following institutions:

BMNH The Natural History Museum (British Museum, Natural History), London, U.K.

CASC California Academy of Sciences, San Francisco, U.S.A.

LACM Natural History Museum of Los Angeles County, Los Angeles, U.S.A.

MCZ Museum of Comparative Zoology, Harvard University, Cambridge, Massachusetts, U.S.A.

MHNG Muséum d’Histoire Naturelle de la Ville de Genève, Geneva, Switzerland

MNHN Muséum National d’Histoire Naturelle, Paris, France

MSNG Museo Civico di Storia Naturale “Giacomo Doria”, Genova, Italy

NHMB Naturhistorisches Museum, Basel, Switzerland

NMK National Museums of Kenya, Nairobi, Kenya

ZFMK Zoological Research Museum Alexander Koenig, Bonn, Germany

## Material and methods

Most of the material examined in this study is located in the Hymenoptera collections of BMNH, CASC, MCZ, MHNG, and LACM. It includes much historical material collected prior to Bolton’s review of *Decamorium* (1976), but the majority of available material has been collected over the past 20 years in a wide range of Afrotropical countries. All new type material and all imaged specimens can be uniquely identified with specimen-level codes affixed to each pin (e.g. CASENT0103295). In the descriptions presented we list all available specimen-level codes for the type series. It should be noted, however, that the number of stated paratype or syntype workers does not necessarily match the number of listed specimen-level codes because pins can sometimes hold more than one specimen, especially for older species. Digital colour montage images were created using a JVC KY-F75 digital camera and Syncroscopy Auto-Montage software (version 5.0), or a Leica DFC 425 camera in combination with the Leica Application Suite software (version 3.8). All images presented are available online and can be seen on AntWeb (http://www.antweb.org). The distribution maps we provide (Figs 61–66) were generated with the R software (R Core Team 2014). We measured 83 workers with a Leica MZ 12.5 equipped with an orthogonal pair of micrometers at a magnification of 100×. Measurements and indices are presented as minimum and maximum values with arithmetic means in parentheses. In addition, all measurements are expressed in mm to two decimal places. The following measurementsand indices used in this study follow Hita Garcia and Fisher (2011, 2012a, 2012b, 2013):

HL Head length: maximum distance from the midpoint of the anterior clypeal margin to the midpoint of the posterior margin of head, measured in full-face view. Impressions on the anterior clypeal margin and the posterior head margin reduce head length.

HW Head width: width of the head directly behind the eyes measured in full-face view.

SL Scape length: maximum scape length excluding basal condyle and neck.

EL Eye length: maximum diameter of compound eye measured in oblique lateral view.

PW Pronotal width: maximum width of the pronotum measured in dorsal view.

WL Weber’s length: diagonal length of the mesosoma in lateral view from the posteroventral margin of propodeal lobe to the anterior-most point of pronotal slope, excluding the neck.

PSL Propodeal spine length: the tip of the measured spine, its base, and the centre of the propodeal concavity between the spines must all be in focus. Using a dual-axis micrometer the spine length is measured from the tip of the spine to a virtual point at its base where the spine axis meets orthogonally with a line leading to the median point of the concavity.

PTH Petiolar node height: maximum height of the petiolar node measured in lateral view from the highest (median) point of the node to the ventral outline. The measuring line is placed at an orthogonal angle to the ventral outline of the node.

PTL Petiolar node length: maximum length of the dorsal face of the petiolar node from the anterodorsal to the posterodorsal angle, measured in dorsal view excluding the peduncle.

PTW Petiolar node width: maximum width of the dorsal face of the petiolar node measured in dorsal view.

PPH Postpetiole height: maximum height of the postpetiole measured in lateral view from the highest (median) point of the node to the ventral outline. The measuring line is placed at an orthogonal angle to the ventral outline of the node.

PPL Postpetiole length: maximum length of the postpetiole measured in dorsal view.

PPW Postpetiole width: maximum width of the postpetiole measured in dorsal view.

OI Ocular index: EL / HW * 100

CI Cephalic index: HW / HL * 100

SI Scape index: SL / HW * 100

DMI Dorsal mesosoma index: PW / WL * 100

LMI Lateral mesosoma index: PH / WL * 100

PSLI Propodeal spine index: PSL / HL * 100

PeNI Petiolar node index: PTW / PW * 100

LPeI Lateral petiole index: PTL / PTH * 100

DPeI Dorsal petiole index: PTW / PTL * 100

PpNI Postpetiolar node index: PPW / PW * 100

LPpI Lateral postpetiole index: PPL / PPH * 100

DPpI Dorsal postpetiole index: PPW / PPL * 100

PPI Postpetiole index: PPW / PTW * 100

Pubescence and pilosity are often of high diagnostic value within the genus *Tetramorium* (e.g. Bolton 1976, 1980, 1985; Hita Garcia et al. 2010; Hita Garcia and Fisher 2012a, 2012b). The varying degree of inclination of pilosity is particularly important for the diagnosis of groups or species. In this context we use the terms “erect”, “suberect”, “subdecumbent”, “decumbent”, and “appressed” following Wilson (1955). The terminology used for the description of surface sculpturing follows Harris (1979) and Bolton (1980).

## Results

### *Tetramorium* Mayr

*Tetramorium* Mayr, 1855: 423. Type species: *Formica caespitum*, by subsequent designation of Girard 1879: 1016.

*Tetrogmus* Roger, 1857: 10. Type species: *Tetrogmus caldarius*, by monotypy. [*Tetrogmus* junior synonym of *Tetramorium*: Roger 1862: 297; Bolton 1976: 359; confirmed here.]

*Xiphomyrmex* Forel, 1887: 385 [as subgenus of *Tetramorium*]. Type species: *Tetramorium (Xiphomyrmex) kelleri*, by subsequent designation of Wheeler, W.M. 1911: 175. [*Xiphomyrmex* junior synonym of *Tetramorium*: Bingham 1903: 175; Bolton 1976: 359; Bolton 1980: 195; Bolton 1994: 106; Bolton 2014; confirmed here].

*Triglyphothrix* Forel, 1890: cvi. Type species: *Triglyphothrix walshi*, by monotypy. [*Triglyphothrix* junior synonym of *Tetramorium*: Bolton 1985: 247; confirmed here.]

*Atopula* Emery, 1912: 104. Type species: *Atopomyrmex nodifer*, by original designation. [*Atopula* junior synonym of *Tetramorium*: Bolton 1976: 359; Bolton 1980: 195; Bolton 1994: 106; confirmed here.]

*Decamorium* Forel, 1913a: 121 [as subgenus of *Tetramorium*]. Type species: *Tetramorium (Decamorium) decem*, by monotypy. [*Decamorium* raised to genus: Emery 1914: 42; Wheeler W.M. 1922: 664, 906.] **Syn. n.**

*Macromischoides* Wheeler, W.M. 1920: 53. Type species: *Macromischa aculeata*, by original designation. [*Macromichoides* Santschi, 1924: 206, incorrect subsequent spelling.] [*Macromischoides* junior synonym of *Tetramorium*: Bolton 1976: 359; Bolton 1980: 196, confirmed here.]

*Lobomyrmex* Kratochvíl, 1941: 84 [as subgenus of *Tetramorium*]. Type species: *Tetramorium (Lobomyrmex) ferox silhavyi* (junior synonym of *Tetramorium ferox*), by monotypy. [*Lobomyrmex* junior synonym of *Tetramorium*: Bolton 1976: 359; Bolton 1980: 196; confirmed here.]

*Sulcomyrmex* Kratochvíl, 1941: 84 [as subgenus of *Tetramorium*]. Unavailable name. Proposed without designation of type species and therefore unavailable. Species included by Kratochvíl (1941) are all referable to *Tetramorium*: Bolton 1976: 359.

*Apomyrmex* Calilung, 2000: 66. Type species: *Apomyrmex manobo*, by original designation. [*Apomyrmex* junior synonym of *Tetramorium*: Bolton 2003: 227, 269; confirmed here.]

***Decamorium* Forel–a junior synonym of *Tetramorium* Mayr**

As outlined in the introduction, in the past various authors expressed very different opinions about the status of *Decamorium*. After examination of all available material and dissemination of all previous literature, we have come to the conclusion that *Decamorium* is best treated as a junior synonym of *Tetramorium*. Our reasons are summarised below:

#### 1. Antennomere count

As outlined above, the antennomere count was the main diagnostic character qualifying *Decamorium* as a genus (Emery 1924; Bolton 1976). Antennomere count has traditionally been considered a very good diagnostic character for separating closely related genera. Yet over the past few decades it has become apparent that the antennal count can vary within a genus, sometimes significantly. Some examples include the genera *Carebara* Westwood with eight to eleven segments (Fernandez 2004), *Temnothorax* Mayr which typically has twelve segments, rarely eleven (Bolton 2003; Radchenko 2004), *Cardiocondyla* Emery with eleven and twelve segments (Seifert 2003), or Pheidole with nine to twelve (Bolton 2003). Also, in some African species of *Carebara* the major workers always have one antennal segment more than the minor workers. Furthermore, subgroups of the same genus often have been placed in different genera in the past due to varying antennomere counts. One good example is *Myrmelachista* Roger outlined in Longino (2006). It was originally described by Roger (1863) as two genera: *Decamera* Roger (a junior homonym of a beetle genus and replaced by the name *Hincksidris* Donisthorpe) having ten-segmented antennae, and *Myrmelachista* having eleven-segmented antennae. This division turned out to be incorrect, and Brown (1973) and Snelling and Hunt (1976) formally synonymised them more than a century later.

In what is now considered to be *Tetramorium* one can find eleven-segmented and twelve-segmented antennae throughout all biogeographical regions, even though most of these forms were previously separated into *Xiphomyrmex* (11-segmented antennae) and *Tetramorium* (12-segmented antennae). Bolton (1976) provided evidence based on sting appendage types showing that this separation was an artificial one, and consequently synonymised *Xiphomyrmex* under *Tetramorium*. Based on this intrageneric variation in antennal segmentation, we accept that a small and highly specialised African species group within *Tetramorium* could have an even more reduced count of ten antennal segments.

This is further supported by the presence of a very small species from India that possesses 10-segmented antennae: *Tetramorium decamerum* (Forel). This species was treated as *Triglyphothrix* by Bolton (1976), thus not taken into consideration as a *Tetramorium*. The later synonymisation of *Triglyphothrix* under *Tetramorium* Bolton (1985) provided a “genuine” *Tetramorium* with 10-segmented antennae. Consequently, this character is not unique to *Decamorium*, but already present in *Tetramorium*.

#### 2. Clypeal shield

**Figure 1.**
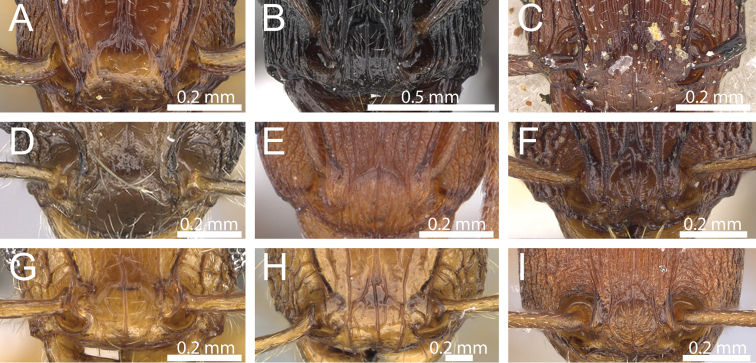
Anterior head showing varying development of the clypeal shield. **A** *Tetramorium decem* (CASENT0914088) **B** *Tetramorium nodiferum* (CASENT0217218) **C** *Tetramorium simulator* (CASENT0914089) **D** *Tetramorium aculeatum* (CASENT0235778) **E** *Tetramorium anodontion* Bolton (CASENT0102334) **F** *Tetramorium diomandei* Bolton (CASENT0901166) **G** *Tetramorium hecate* Hita Garcia & Fisher (CASENT0248334) **H** *Tetramorium melanogyna* Mann (CASENT0199931) **I** *Tetramorium sericeiventre* (CASENT0235773).

The reduced clypeal shield seen in *Decamorium* (Fig. 1A) is not unique to its species. Within the tropical *Tetramorium* fauna most species have a very well-developed and clearly distinctive clypeal shield (Figs 1F, 1G, 1F, 1I), but there are a number of species, such as *Tetramorium nodiferum* (Emery) (Fig. 1B), *Tetramorium simulator* Arnold (Fig. 1C), *Tetramorium aculeatum* (Mayr) (Fig. 1D), or *Tetramorium anodontion* Bolton (Fig. 1E), in which this shield is much less pronounced or almost reduced. The clypeal shield generally varies from species to species in its height and the sharpness of its dorsal edge. When the development of this character across several hundred *Tetramorium* species is considered, *Decamorium* emerges as one extreme of a cline that ranges from almost no clypeal shield to a very sharp and high shield, such as in the members of the *Tetramorium sericeiventre* Emery species group (Fig. 1I).

#### 3. Mandibular dentition

The mandibular dentition of *Decamorium* and *Tetramorium* seemed slightly different back in 1976, but as anticipated by Bolton, it has become clear that there is much more variation within *Tetramorium*. Currently there is no significant difference in mandibular dentition between *Decamorium* and *Tetramorium*. In *Decamorium* the mandibular count consists of three apical teeth followed by a series of four or five denticles, while in *Tetramorium* there are two to three apical teeth followed by a series of three to eight denticles (Bolton 2003). Consequently, this character has no diagnostic importance in this group since the values of *Decamorium* fall well within the range of the larger *Tetramorium*.

#### 4. *Tetramorium simulator* Arnold

**Figure 2.**
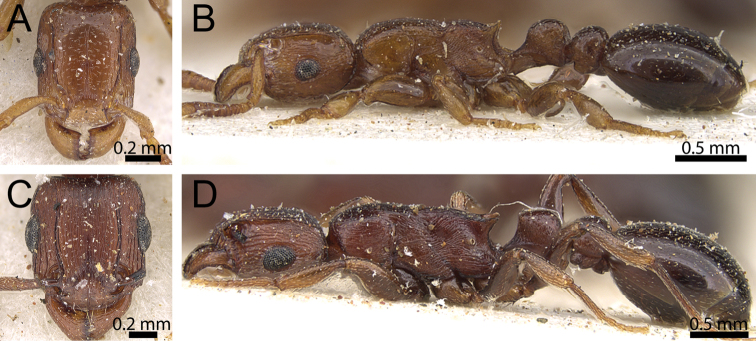
Head in full-face view and body in profile. **A, B** *Tetramorium decem* (CASENT0914087) **C, D** *Tetramorium simulator* (CASENT0914089).

If one considers the whole tribe Tetramoriini, then it becomes apparent that the specialised habitus of *Decamorium* is not unique. Several authors have stated that *Decamorium* are specialised termite hunters, and that their specialised morphology could be an adaptation to such a dangerous lifestyle (Arnold 1917; Bolton 1976; Longhurst et al. 1979). Interestingly, both Arnold (1917) in the original description and later Bolton (1980) noted the similarities in general body shape and diet between members of *Decamorium* and the species *Tetramorium simulator* from South Africa. We agree that the similarities in morphology are indeed obvious, especially in profile view (Fig. 2). However, at present it is not clear whether the shared morphology is based on a close phylogenetic relationship between *Decamorium* and *Tetramorium simulator* or a result of convergent evolution due to a similar lifestyle hunting termites. We believe the latter more likely since the twelve-segmented antennae, the much broader head, and sculptured clypeus of *Tetramorium simulator* suggest a closer relationship to another group with twelve-segmented antennae than to *Decamorium*. Therefore, we hypothesise that both have evolved from different *Tetramorium* lineages and acquired the specialised habitus independently from each other. Another remarkable aspect is the lack of a strong and sharp clypeal shield in *Tetramorium simulator*, which seems to have been reduced in a manner almost similar, though less pronounced, to *Decamorium*.

#### 5. Male morphology

We do not intend to go into details of male morphology here, but so far there is not a single character that would separate the males of *Decamorium* from the males of *Tetramorium*; a result that agrees with Bolton’s findings (1976). It should be noted, however, that *Decamorium* males are very rare, and only one specimen was available for examination (BMNH: CASENT0901037).

#### 6. Molecular evidence

In addition to our morphological analysis above, there is also molecular evidence supporting the synonymisation of *Decamorium* under *Tetramorium*. Based on a multi-gene dataset, Ward et al. (in press) show that *Decamorium* is nested within a larger *Tetramorium* clade. However, how *Decamorium* is integrated into *Tetramorium* and to which groups/lineages it is most closely related remains unknown. Further phylogenetic/phylogenomic studies that deal with a greater number of species groups and a good proportion of species are needed to clarify relationships within the hyperdiverse *Tetramorium* and its satellite genera.

## Revision of the *Tetramorium decem* species group

### Synopsis of the *Tetramorium decem* species group

*Tetramorium decem* Forel, 1913a, **comb. r.**

*Tetramorium raptor* Hita Garcia, **sp. n.**

*Tetramorium uelense* Santschi, 1923, **comb. r.**

*Tetramorium ultor* Forel, 1913b, **comb. r., stat. r. & stat. n.**

*Tetramorium venator* Hita Garcia, **sp. n.**

### Diagnosis of *Tetramorium decem* species group

Ten-segmented antennae; antennal scape relatively short (SI 67–76); anterior clypeal margin with distinct but often shallow impression; frontal carinae strongly developed and noticeably raised, forming dorsal margin of very well-developed antennal scrobes, curving down ventrally and anteriorly halfway between posterior eye margin and posterior head margin and forming posterior and usually ventral scrobe margins; antennal scrobes very well developed, deep and usually with clearly defined margins all around, median scrobal carina absent; eyes relatively large (OI 32–40); mesosoma relatively flat, low, and elongated, margination between lateral and dorsal mesosoma moderately developed (LMI 33–38); propodeum armed with short triangular to elongate-triangular teeth (PSLI 9–19); propodeal lobes short, rounded to triangular; tibiae and femorae strongly swollen; petiolar node nodiform with moderately rounded antero- and posterodorsal margins, petiolar dorsum weakly to strongly convex, node in profile between 1.0 to 1.3 times higher than long (LPeI 77–100), node in dorsal view around 1.1 to 1.3 times longer than wide (DPeI 76–92); postpetiole in profile globular, around 1.1 to 1.4 times higher than long (LPpI 71–88); mandibles and clypeus unsculptured, smooth, and shiny; sculpture on cephalic dorsum between frontal carinae and dorsal mesosoma variable, ranging from unsculptured, smooth, and shiny to longitudinally rugose/rugulose, often punctate or puncticulate; petiole usually weakly sculptured, postpetiole unsculptured to weakly sculptured; gaster unsculptured, smooth, and shiny; pilosity greatly reduced, head with several pairs of standing hairs, mesosoma with one pair, waist segments sometimes with one long pair each, and sometimes first gastral tergite with one pair; sting appendage triangular.

### Taxonomic and biogeographic notes on the group

**Figure 3.**
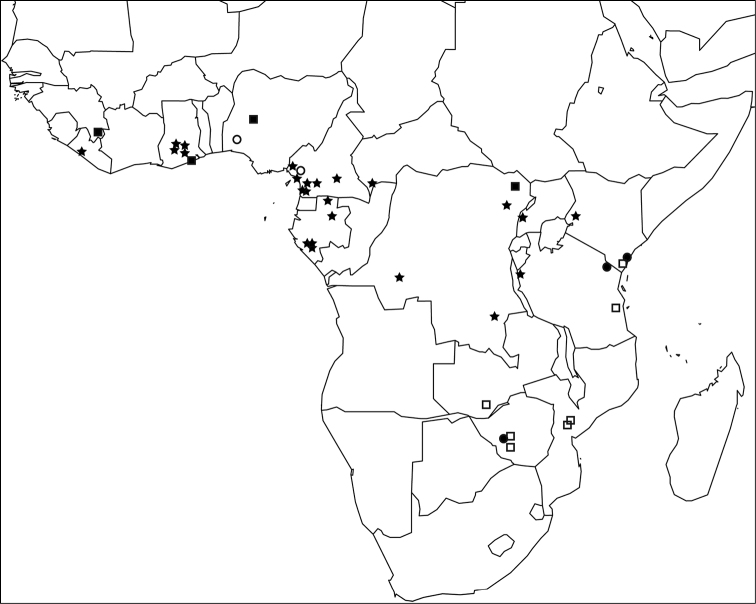
Map of sub-Saharan Africa showing the known distribution ranges of the five members of the *Tetramorium decem* species group: *Tetramorium decem* (filled circle), *Tetramorium raptor* (empty circle), *Tetramorium uelense* (filled square), *Tetramorium ultor* (empty square), and *Tetramorium venator* (star).

The *Tetramorium decem* species group is endemic to the Afrotropical region where it is widely distributed (Fig. 3). *Tetramorium raptor* and *Tetramorium uelense* are found in West and Central Africa and *Tetramorium venator* occurs through most of the equatorial rainforest belt from Liberia in West Africa to Western Kenya. By contrast, *Tetramorium decem* and *Tetramorium ultor* are species from eastern and southeastern Africa. Surprisingly, the group seems to be absent from South Africa based on the material available to us, but *Tetramorium decem* or *Tetramorium ultor* are likely to be found there or in neighbouring Botswana or Namibia. Furthermore, we expect the distribution ranges of *Tetramorium decem*, *Tetramorium uelense*, and perhaps *Tetramorium ultor* to expand with further ant inventory or collecting projects in Afrotropical savannahs, dry forests, and other arid habitats. These were sparsely sampled in sub-Saharan Africa in the past since most modern ant inventories have focused on rainforests (e.g. Belshaw and Bolton 1994; Watt et al. 2002; Fisher 2004; Yanoviak et al. 2007; Hita Garcia et al. 2009), whereas only a few studies have examined ant faunas from drier localities (e.g. Robertson 1999, 2002; Braet and Taylor 2008).

The separation of the *Tetramorium decem* species group from all other *Tetramorium* species groups is straightforward and easy. So far, only the members of the *Tetramorium decem* group have ten-segmented antennae, whereas all other Afrotropical *Tetramorium* have either eleven or twelve. Consequently, the *Tetramorium decem* species group is unlikely to be confused with another Afrotropical group. The morphology of the five species of the group is very uniform, likely due to their strongly specialised lifestyle, which makes the taxonomy of the group challenging at first sight. However, good diagnostic characters separate them fairly well from each other, especially eye size, propodeal spine/teeth length, petiolar node shape, mesosomal sculpture, and body colouration. These characters are remarkably consistent within each species throughout its whole distribution, as are the species-specific habitat preferences.

### Identification key for *Tetramorium decem* species group (workers)

**Table:** 

1	Dorsum of promesonotum with conspicuous longitudinally rugose/rugulose sculpture (Fig. 4A, B)	2
–	Dorsum of promesonotum unsculptured, smooth, and usually very shiny (Fig. 4C, D)	3
2	Slightly smaller species (WL 0.88–0.93); propodeum armed with shorter, triangular, and acute teeth (PSLI 10–11); dorsum of promesonotum longitudinally rugulose with very little ground sculpture, lateral pronotum mostly unsculptured and shiny, only dorsally longitudinally rugulose; generally of uniform dark brown colour; rainforest species (Fig. 5A, B) [Cameroon, Nigeria]	*Tetramorium raptor*
–	Slightly larger species (WL 0.98–1.06); propodeum armed with longer, triangular to elongate-triangular, and acute teeth (PSLI 16–18); dorsum of promesonotum and lateral pronotum strongly longitudinally rugose with distinct punctate ground sculpture; strongly bicoloured species with dark brown or black gaster contrasting with light brown to reddish brown on remainder of body; savannah species (Fig. 5C, D) [Cameroon, Ghana, Guinea, Nigeria, and Republic of the Congo]	*Tetramorium uelense*
3	Generally larger species (WL 1.02–1.16); propodeal teeth relatively longer (PSLI 17–19); petiolar node in profile relatively higher, in profile 1.2 to 1.3 times higher than long (LPeI 77–82); strongly bicoloured species with dark brown or black gaster contrasting with light brown to reddish brown remainder of body (Fig. 6A) [Kenya, Tanzania, and Zimbabwe]	*Tetramorium decem*
–	Generally smaller species (WL 0.85–0.98); propodeal teeth relatively shorter (PSLI 9–13); petiolar node relatively lower, in profile around 1.0 to 1.2 times higher than long (LPeI 86–100); usually of uniform brown colour, if bicoloured, then only slightly so and never as well developed as above (Fig. 6B)	4
4	Smaller eyes (OI 33–36); body colouration uniformly light brown to chestnut brown (Fig. 7A, B) [Kenya, Mozambique, Tanzania, Zambia, and Zimbabwe]	*Tetramorium ultor*
–	Larger eyes (OI 37–40); body colouration uniformly dark brown to black, always darker than above (Fig. 7C, D) [Central African Republic, Cameroon, Democratic Republic of Congo, Gabon, Ghana, Kenya, Liberia, Tanzania, Uganda]	*Tetramorium venator*

**Figure 4.**
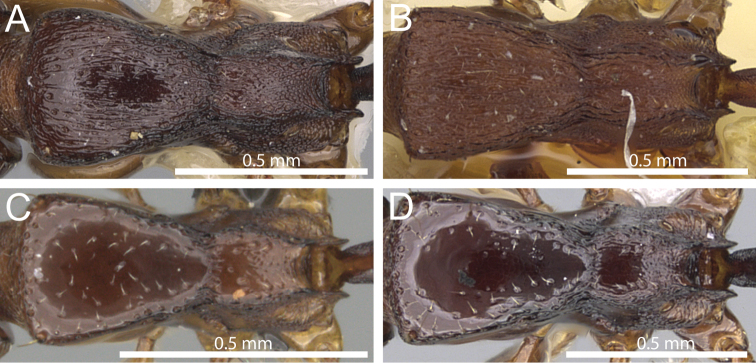
Mesosoma in dorsal view. **A** *Tetramorium raptor* (CASENT0195628) **B** *Tetramorium uelense* (CASENT0914084) **C** *Tetramorium ultor* (CASENT0235465) **D** *Tetramorium venator* (CASENT0401714).

**Figure 5.**
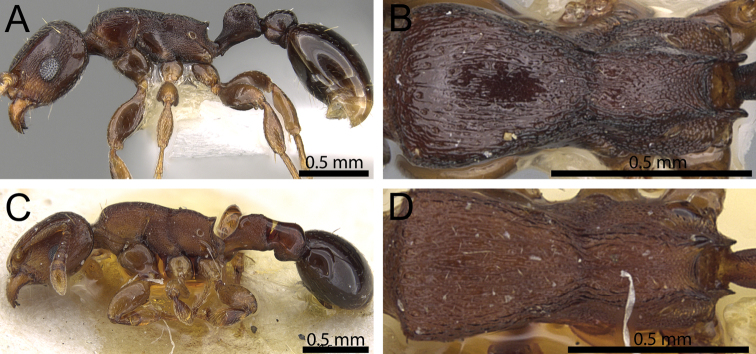
Body in profile and mesosoma in dorsal view. **A, B** *Tetramorium raptor* (CASENT0280848) **C, D** *Tetramorium uelense* (CASENT0914084).

**Figure 6.**
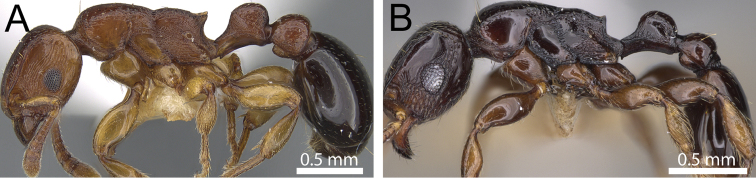
Body in profile. **A** *Tetramorium decem* (CASENT0914088) **B** *Tetramorium venator* (CASENT0195574).

**Figure 7.**
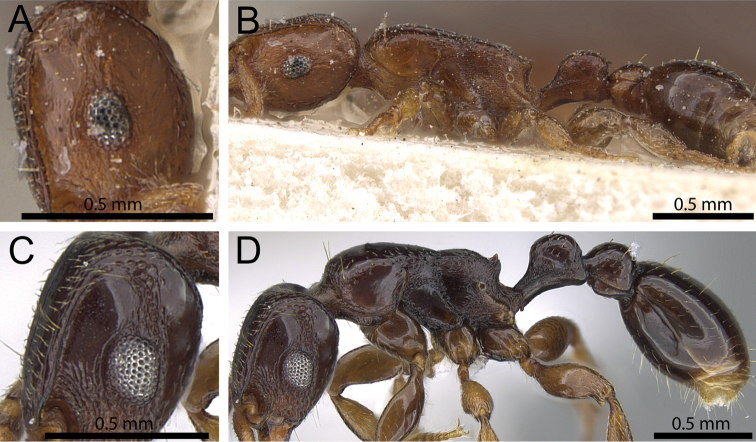
Head and body in profile. **A, B** *Tetramorium ultor* (CASENT0235465) **C, D** *Tetramorium venator* (CASENT0401714).

### Tetramorium decem

Forel, 1913a comb. r.

**Figure 8.**
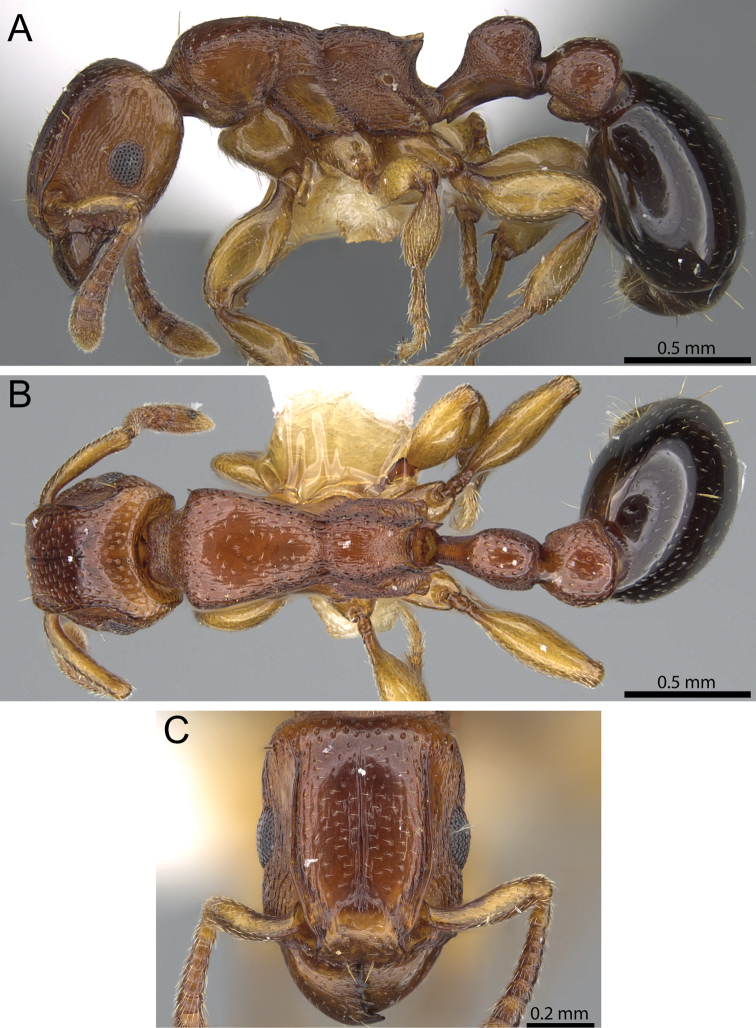
*Tetramorium decem* non-type worker (CASENT0914088). **A** Body in profile **B** Body in dorsal view **C** Head in full-face view.

Figs 1A , 2A , 2B , 3 , 6A , 8

Tetramorium (Decamorium) decem Forel, 1913a: 121. [Combination in *Decamorium* by Wheeler 1922: 906; senior synonym of *Decamorium ultor* by Bolton 1976: 298.]

#### Type material.

**Lectotype** [designated here], pinned worker, ZIMBABWE, Redbank, 19.98333S, 28.37759E, 7.IV.1912 (*G. Arnold*) (MHNG: CASENT0909196) [examined]. **Paralectotypes** [designated here], seven pinned workers with same data as lectotype (BMNH: CASENT0901035; MHNG: CASENT0248316; MSNG: CASENT0904789) [examined].

[Note: the GPS data of the type locality was not provided by the locality label or the original description. The data presented above is based on our own geo-referencing of the town of Redbank located in the Matabeleland North Province. Consequently, the location should be considered as an approximation and not the exact position of the type locality.]

#### Non-type material.

KENYA: Coastal Province, Malindi District, Arabuko Sokoke Forest, 3.28S, 39.97E, 75 m, Brachystegia forest, 26.V.2001 (*R.R. Snelling & D.J. Martins*); Coastal Province, Malindi District, Arabuko Sokoke Forest, 3.32111S, 39.92944E, ca. 50 m, VI.2009 (*F. Hita Garcia & G. Fischer*); TANZANIA: Mkomazi Game Reserve, Ibaya, 3.96667S, 37.8E, in burnt grassland, 19.–20.XI.1994 (*A. Russel-Smith*).

#### Diagnosis.

*Tetramorium decem* can be recognised by the following combination of characters: relatively larger species (HW 0.59–0.62; WL 1.02–1.16); propodeal teeth relatively longer (PSLI 17–19); petiolar node in profile around 1.2 to 1.3 times higher than long (LPeI 77–82); dorsum of promesonotum unsculptured, smooth, and very shiny; strongly bicoloured species with dark brown or black gaster contrasting with light brown to reddish brown remainder of body.

#### Worker measurements

**(N=15).** HL 0.71–0.74 (0.72); HW 0.59–0.62 (0.60); SL 0.42–0.45 (0.43); EL 0.19–0.21 (0.20); PH 0.33–0.37 (0.35); PW 0.47–0.50 (0.48); WL 1.02–1.16 (1.06); PSL 0.12–0.14 (0.13); PTL 0.25–0.27 (0.26); PTH 0.31–0.34 (0.33); PTW 0.22–0.24 (0.23); PPL 0.24–0.27 (0.25); PPH 0.32–0.36 (0.34); PPW 0.32–0.36 (0.34); CI 83–85 (84); SI 70–76 (72); OI 32–34 (33); DMI 41–47 (45); LMI 32–34 (33); PSLI 17–19 (18); PeNI 46–51 (48); LPeI 77–82 (80); DPeI 85–92 (88); PpNI 67–76 (70); LPpI 71–77 (75); DPpI 128–138 (133); PPI 143–149 (147).

#### Worker description.

Head much longer than wide (CI 83–85); posterior head margin weakly concave. Anterior clypeal margin with distinct, but often shallow median impression. Frontal carinae strongly developed and noticeably raised forming dorsal margin of very well-developed antennal scrobes, curving down ventrally and anteriorly halfway between posterior eye margin and posterior head margin and forming posterior and parts of ventral scrobe margins; antennal scrobes very well developed, deep and with clearly defined margins, but ventral margin less strongly developed, median scrobal carina absent. Antennal scapes short, not reaching posterior head margin (SI 70–76). Eyes very large (OI 32–34). Mesosomal outline in profile flat to weakly convex, relatively low and elongate (LMI 32–34), moderately to strongly marginate from lateral to dorsal mesosoma; promesonotal suture absent; metanotal groove present, distinct, and clearly impressed. Propodeal spines short, elongate-triangular, and moderately acute (PSLI 17–19), propodeal lobes short, triangular, and usually blunt, always significantly shorter than propodeal spines. Tibiae and femorae strongly swollen. Petiolar node nodiform with moderately rounded antero- and posterodorsal margins, around 1.2 to 1.3 times higher than long (LPeI 77–82), anterior and posterior faces approximately parallel, anterodorsal and posterodorsal margins situated at about the same height, petiolar dorsum clearly convex; node in dorsal view between 1.1 to 1.2 times longer than wide (DPeI 85–92), in dorsal view pronotum around 2.0 to 2.2 times wider than petiolar node (PeNI 46–51). Postpetiole in profile globular to subglobular, approximately 1.3 to 1.4 times higher than long (LPpI 71–77); in dorsal view around 1.3 to 1.4 times wider than long (DPpI 128–138), pronotum between 1.3 to 1.5 times wider than postpetiole (PpNI 67–76). Postpetiole in profile usually appearing less voluminous than petiolar node, postpetiole in dorsal view around 1.4 to 1.5 times wider than petiolar node (PPI 143–149). Mandibles and clypeus usually fully unsculptured, smooth, and shining, mandibles sometimes with few traces of rugulae apically; cephalic dorsum between frontal carinae mostly unsculptured and shiny, median ruga present and distinct, cephalic dorsum also puncticulate to punctate throughout its length, posteriorly close to posterior head margin especially pronounced; scrobal area partly unsculptured, smooth and shiny and partly merging with surrounding rugose sculpture on sides of head. Ground sculpture on head usually weak to absent. Dorsum of mesosoma mostly unsculptured, smooth and shiny with scattered punctures, rarely with few traces of rugulae; lateral mesosoma longitudinally rugose and very conspicuously reticulate-punctate except for mostly unsculptured lateral pronotum and katepisternum. Forecoxae unsculptured, smooth, and shining. Petiolar node and postpetiole superficially longitudinally rugulose or irregularly rugulose superimposed on conspicuous but relatively weak reticulate-punctate ground sculpture. Mesosoma and waist segments appearing mostly matt. First gastral tergite unsculptured, smooth, and shiny. Pilosity and pubescence greatly reduced: head with few pairs of moderately long, standing hairs, anterior pronotum with one long pair, waist segments sometimes with one long pair each, and sometimes first gastral tergite with one long pair; appressed pubescence present everywhere on body, but noticeable only on antennae, cephalic dorsum, legs, and first gastral tergite. Anterior edges of antennal scapes and dorsal (outer) surfaces of hind tibiae with appressed hairs. Body strongly bicoloured with dark brown to black gaster contrasting with light brown to reddish brown remainder.

#### Distribution and biology.

The distribution range of *Tetramorium decem* is far smaller than previously thought (Fig. 3). Indeed, most of the material listed in the literature as *Tetramorium decem* or labelled as such in museum collections turned out to be either *Tetramorium ultor* or *Tetramorium venator*, while only a few collections proved to be genuine *Tetramorium decem*. Based on the redefined species definition, *Tetramorium decem* is only known from the type locality in Zimbabwe and two additional localities in East Africa: Arabuko Sokoke in Kenya and Mkomazi in Tanzania. Nevertheless, if more extensive sampling efforts are undertaken in East Africa, *Tetramorium decem* is likely to be found in more localities in Kenya, Tanzania, and Zimbabwe. Like *Tetramorium uelense* and *Tetramorium ultor*, *Tetramorium decem* prefers arid habitats, such as savannah and woodland. Based on Arnold (1917) and the collection label from some material from Arabuko Sokoke, *Tetramorium decem* nests in sandy soil. The diet consists of termites, as with most other members of the species group.

#### Discussion.

*Tetramorium decem* is the core species of the group, and was the type species for the description of the subgenus *Decamorium* by Forel (1913a). It is perhaps the most conspicuous species of the group. Its bicolouration, larger size, lack of sculpture on the mesosomal dorsum, and a higher petiolar node render it immediately recognisable. The mostly unsculptured, smooth and shiny mesosomal dorsum distinguishes *Tetramorium decem* from *Tetramorium raptor* and *Tetramorium uelense*, in which the dorsum of the mesosoma is clearly longitudinally rugose/rugulose. *Tetramorium ultor* and *Tetramorium venator* both share the lack of sculpture on the mesosomal dorsum with *Tetramorium decem*, but can still be easily separated from the latter. *Tetramorium decem* is generally larger in size (WL 1.02–1.16), has longer propodeal spines (PSLI 17–19) and is also conspicuously bicoloured, whereas *Tetramorium ultor* and *Tetramorium venator* are smaller species (WL 0.85–0.98) with significantly shorter propodeal teeth (PSLI 9–13) and a more uniform brown to black body colouration. In addition, *Tetramorium decem* also has a higher petiolar node, in profile around 1.2 to 1.3 times higher than long (LPeI 77–82), compared to the other two, in which the node in profile is only around 1.0 to 1.2 times higher than long (LPeI 86–100). The species that appears to be morphologically closest to *Tetramorium decem* is *Tetramorium uelense*. Both species share the large body, bicolouration, and preference for arid habitats. However, in addition to the sculpture on the mesosoma, *Tetramorium uelense* also has a lower petiolar node, in profile around 1.1 times higher than long (LPeI 88–93). Another character that is shared between *Tetramorium decem* and *Tetramorium uelense* but absent in the other species of the group is the development of the ventral margin of the antennal scrobe. In *Tetramorium raptor*, *Tetramorium ultor*, and *Tetramorium venator* the margin is clearly and well defined, while in *Tetramorium decem* and *Tetramorium uelense* it is less so and merges more with the surrounding rugose sculpture.

#### Variation.

Based on the available material we did not observe any significant form of intraspecific variation in *Tetramorium decem*.

### Tetramorium raptor

Hita Garcia sp. n.

http://zoobank.org/6A9F212B-8460-41C0-9F8C-792D9A4780C4

http://species-id.net/wiki/Tetramorium_raptor

Figs 3 , 4A , 5 , 9

#### Type material.

**Holotype**, pinned worker, CAMEROON, Sud-Ouest, Bakundu, 4.49222N, 9.375E, collection code ANTC27989, 8.XI.1990 (*A. Dejean*) (BMNH: CASENT0195628). **Paratypes**, 14 pinned workers with same data as holotype (BMNH: CASENT0195581; CASENT0195630; CASENT0195631; CASC: CASENT0195633; CASENT0195634; LACM: LACM_ENT_323500; MCZ: CASENT0195628; ZFMK: CASENT0195632).

**Figure 9.**
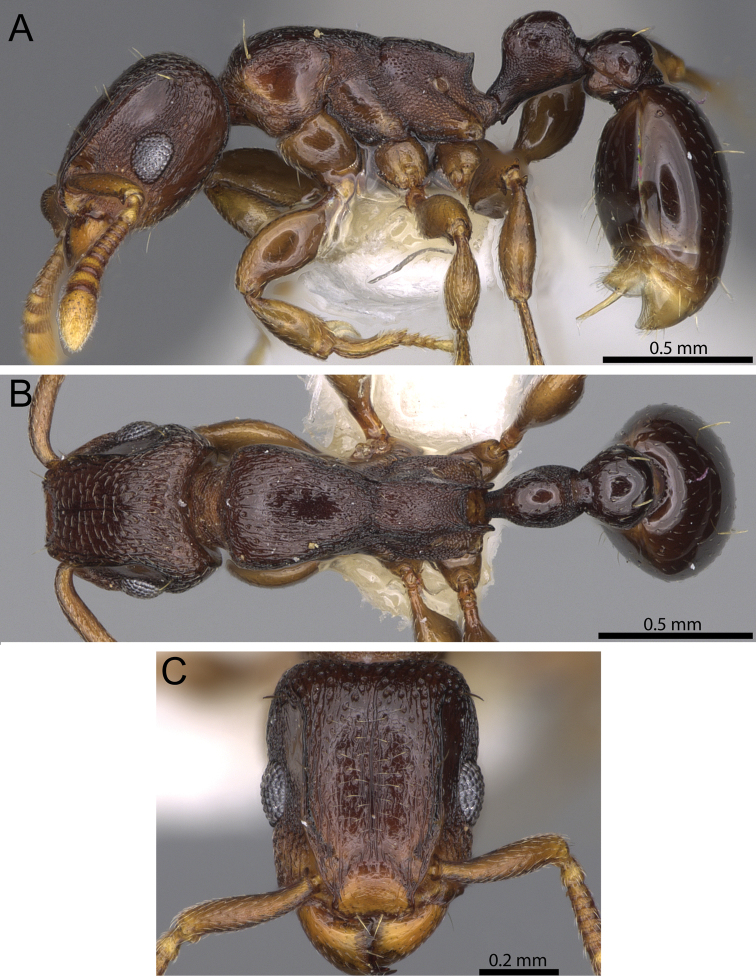
*Tetramorium raptor* holotype worker (CASENT0195628). **A** Body in profile **B** Body in dorsal view **C** Head in full-face view.

[Note: the GPS data of the type locality was not provided by the locality label. The data presented above is based on our own geo-referencing of the Bakundu Forest located in the province Sud-Ouest. Consequently, it should be considered an approximation and not the exact position of the type locality.]

#### Non-type material.

CAMEROON: Sud-Ouest, Bakundu, 4.49222N, 9.375E, 8.XI.1990 (*A. Dejean*); NIGERIA: Gambari, 10.VI.1969 (*B. Bolton*); Gambari, C.R.I.N., 17.VI.1975 (*B. Taylor*).

#### Diagnosis.

*Tetramorium raptor* is easily recognisable within the group on the basis of the following combination of characters: relatively smaller species (WL 0.88–0.93); very large eyes (OI 35); propodeum armed with very short triangular teeth (PSLI 10–11); petiolar node in profile around 1.1 times higher than long (LPeI 89–93); dorsum of mesosoma with longitudinally rugulose sculpture; body uniformly dark brown, appendages of lighter brown.

#### Worker measurements

**(N=12).** HL 0.64–0.68 (0.67); HW 0.53–0.56 (0.54); SL 0.37–0.41 (0.39); EL 0.19–0.20 (0.XX); PH 0.31––0.34 (0.33); PW 0.40–0.43 (0.41); WL 0.88–0.93 (0.91); PSL 0.07–0.08 (0.07); PTL 0.23–0.25 (0.24); PTH 0.26–0.28 (0.27); PTW 0.19–0.21 (0.20); PPL 0.21–0.24 (0.22); PPH 0.25–0.28 (0.27); PPW 0.26–0.30 (0.28); CI 80–83 (82); SI 70–73 (72); OI 35; DMI 44–47 (45); LMI 35–37 (36); PSLI 10–11 (11); PeNI 47–51 (48); LPeI 89–93 (90); DPeI 80–85 (82); PpNI 64–70 (68); LPpI 81–88 (85); DPpI 123–130 (125); PPI 137–150 (142).

#### Worker description.

Head much longer than wide (CI 80–83); posterior head margin weakly concave. Anterior clypeal margin with distinct but often shallow median impression. Frontal carinae strongly developed and noticeably raised forming dorsal margin of very well-developed antennal scrobes, curving down ventrally and anteriorly halfway between posterior eye margin and posterior head margin and forming posterior and ventral scrobe margins; antennal scrobes very well developed, deep and with clearly defined margins all around, median scrobal carina absent. Antennal scapes short, far from reaching posterior head margin (SI 70–73). Eyes relatively large (OI 35). Mesosomal outline in profile relatively flat, elongate and low (LMI 35–37), moderately to strongly marginate from lateral to dorsal mesosoma; promesonotal suture absent; metanotal groove present and conspicuous, but relatively shallow. Propodeum armed with short, triangular, and usually acute teeth (PSLI 10–11), propodeal lobes short, well rounded, and usually larger than propodeal teeth. Petiolar node nodiform with moderately rounded antero- and posterodorsal margins, in profile around 1.1 times higher than long (LPeI 89–93), anterior and posterior faces approximately parallel, anterodorsal and posterodorsal margins situated at about same height and equally angled, petiolar dorsum weakly convex; node in dorsal view around 1.2 to 1.3 times longer than wide (DPeI 80–85), in dorsal view pronotum around 2.0 to 2.2 times wider than petiolar node (PeNI 47–51). Postpetiole in profile globular, approximately 1.1 to 1.2 times higher than long (LPpI 81–88); in dorsal view around 1.2 and 1.3 times wider than long (DPpI 123–130), pronotum around 1.4 to 1.6 times wider than postpetiole (PpNI 64–70). Postpetiole in profile appearing less voluminous than petiolar node, postpetiole in dorsal view around 1.4 to 1.5 times wider than petiolar node (PPI 137–150). Mandibles and clypeus unsculptured, smooth, and shining; cephalic dorsum between frontal carinae with fine irregularly longitudinally rugulose sculpture, rugulae running from posterior clypeal margin to posterior head margin, often interrupted or meandering, rarely with cross-meshes, cephalic dorsum also puncticulate to punctate throughout its length, otherwise without ground sculptured; scrobal area partly unsculptured, smooth and shiny and partly strongly reticulate-punctate; lateral head mainly reticulate-rugose with weak to moderately well developed punctate ground sculpture. Dorsum of mesosoma densely longitudinally rugulose, anteriorly without much ground sculpture, posteriorly on top of strong reticulate-punctate ground sculpture; lateral pronotum and katepisternum mostly unsculptured, smooth, and shiny, remainder of lateral mesosoma irregularly rugose and very conspicuously reticulate-punctate. Forecoxae unsculptured, smooth, and shining. Petiolar node laterally reticulate-punctate, dorsum of node mostly unsculptured, smooth, and shiny; postpetiole mostly unsculptured, smooth, and shiny with scattered punctures. First gastral tergite unsculptured, smooth, and shiny. Pilosity and pubescence greatly reduced: head with few pairs of moderately long, standing hairs, anterior pronotum with one long pair, waist segments sometimes with one long pair each, and sometimes first gastral tergite with one long pair; appressed pubescence present everywhere on body, but noticeable only on antennae, cephalic dorsum, legs, and first gastral tergite. Anterior edges of antennal scapes and dorsal (outer) surfaces of hind tibiae with appressed hairs. Body uniformly dark brown to black, appendages of lighter brown.

#### Etymology.

The name of the new species is Latin and means “thief, robber, or plunderer”. It refers to the predaceous lifestyle of *Tetramorium raptor*. The species epithet is a nominative noun, and thus invariant.

#### Distribution and biology.

*Tetramorium raptor* is currently only known from the type locality Bakundu in the southeast of Cameroon and from Gambari in south-western Nigeria (Fig. 3). Based on the minimal collection label data, *Tetramorium raptor* lives in rainforest leaf litter.

#### Discussion.

*Tetramorium raptor* is an easily distinguishable member of the *Tetramorium decem* group, but was not recognised until this study. Indeed, all known material was collected in 1969 and 1990, but labelled as *Tetramorium uelense* on the basis of the distinctive sculpture on the mesosomal dorsum. The presence of conspicuous, longitudinally rugulose sculpture on the dorsum of the promesonotum distinguishes it from *Tetramorium decem*, *Tetramorium ultor*, or *Tetramorium venator*, since the latter three all lack sculpture on the promesonotal dorsum. *Tetramorium uelense*, however, shares the presence of sculpture on the mesosomal dorsum with *Tetramorium raptor*, which led to the abovementioned misidentifications. Nevertheless, careful examination of all material previously listed as *Tetramorium uelense* revealed the presence of two morphologically and ecologically different species. The most obvious differences are body size and colour. *Tetramorium uelense* is strongly bicoloured and larger (WL 0.98–1.06) than the smaller and uniformly-coloured *Tetramorium raptor* (WL 0.88–0.93). The latter also has shorter propodeal teeth (PSLI 10–11) than *Tetramorium uelense* (PSLI 16–18). Furthermore, *Tetramorium raptor* possesses a longitudinally rugulose promesonotal dorsum with very little ground sculpture and a mostly unsculptured and shiny lateral pronotum, whereas *Tetramorium uelense* has a promesonotal dorsum that is longitudinally rugose with distinct punctate ground sculpture and a lateral pronotum that is conspicuously rugose with prominent ground sculpture. In addition, both species also differ in habitat choice, as *Tetramorium uelense* seems to prefer savannah while *Tetramorium raptor* lives in rainforest.

#### Variation.

Based on material from the two known localities, there is no intraspecific variation in *Tetramorium raptor*.

### Tetramorium uelense

Santschi, 1923 comb. r.

**Figure 10.**
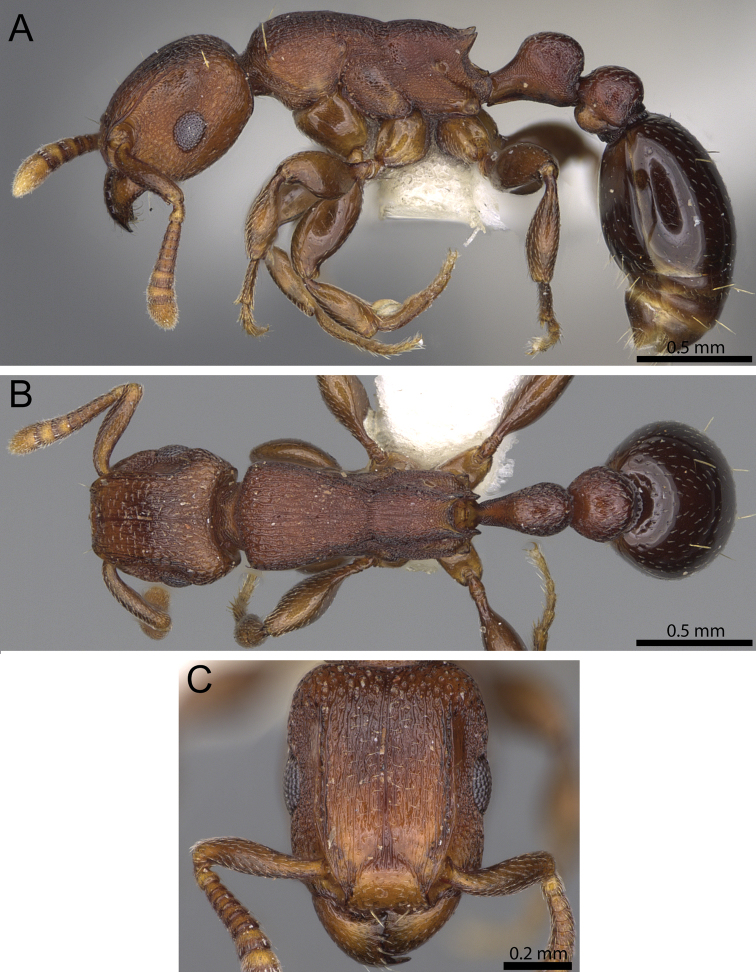
*Tetramorium uelense* non-type worker (CASENT0195580). **A** Body in profile **B** Body in dorsal view **C** Head in full-face view.

Figs 3 , 4B , 5C , 5D , 10

Tetramorium (Decamorium) decem uelense Santschi, 1923: 285. [Combination in *Decamorium* and raised to species by Bolton 1976: 298.]
Decamorium decem nimba Bernard, 1953: 250. [Junior synonym of *Tetramorium uelense* by Bolton 1976: 298; here confirmed.]

#### Type material.

**Of *uelense*:** **lectotype**, pinned worker, D. R. CONGO (Congo belge), Uelé, Vankerhovenville, 3.0N, 29.5E (*Degreef*) (NHMB: CASENT0906826) [examined]. **Paralectotype**, pinned queen with same data as lectotype (MRAC) [not examined].

**Of *nimba*:** **holotype**, pinned worker, GUINEA, Kéoulenta, 7.714053N, 8.331786W, St. 1 Savane, (MNHN: CASENT0914084) [examined].

[Note: GPS data for neither of the type localities was included on the locality labels or the original descriptions. The data presented above is based on our own geo-referencing of Vankerhovenville located in Province Orientale and Kéoulenta located in the Nzérékoré Region. Consequently, they should be considered approximations and not the exact positions of the type localities.]

#### Non-type material.

GHANA: Greater Accra Region, Accra Metropolis District, Legon, 23.VIII.1972 (*D. Leston*); NIGERIA: 16 km N. of Mokwa, 16.X.1976 (*C. Longhurst*).

#### Diagnosis.

The following character combination separates *Tetramorium uelense* from the other species of the *Tetramorium decem* species group: relatively larger species (WL 0.98–1.06); propodeum armed with short triangular to elongate-triangular teeth (PSLI 16–18); petiolar node in profile around 1.1 times higher than long (LPeI 88–93); dorsum of mesosoma conspicuously longitudinally rugose with distinctive reticulate-punctate ground sculpture; strongly bicoloured with dark brown to black gaster contrasting with light brown to reddish brown remainder of body.

#### Worker measurements

**(N=6).** HL 0.67–0.72 (0.70); HW 0.54–0.59 (0.57); SL 0.39–0.42 (0.41); EL 0.19–0.20 (0.20); PH 0.36–0.38 (0.37); PW 0.43–0.47 (0.45); WL 0.98–1.06 (1.02); PSL 0.11–0.13 (0.10); PTL 0.27–0.29 (0.28); PTH 0.29–0.32 (0.31); PTW 0.21–0.23 (0.22); PPL 0.24–0.26 (0.25); PPH 0.28–0.34 (0.31); PPW 0.30–0.33 (0.31); CI 80–83 (81); SI 69–74 (72); OI 34–35 (35); DMI 43–44 (44); LMI 35–37 (36); PSLI 16–18 (17); PeNI 48–49 (49); LPeI 88–93 (90); DPeI 77–81 (79); PpNI 69–70 (70); LPpI 75–86 (80); DPpI 122–125 (124); PPI 141–145 (143).

#### Worker description.

Head much longer than wide (CI 80–83); posterior head margin weakly concave. Anterior clypeal margin with distinct, but often shallow median impression. Frontal carinae strongly developed and noticeably raised forming dorsal margin of very well-developed antennal scrobes, curving down ventrally and anteriorly halfway between posterior eye margin and posterior head margin and forming posterior and ventral scrobe margins; antennal scrobes very well developed, deep and with clearly defined margins, but ventral margin less strongly developed, median scrobal carina absent. Antennal scapes short, far from reaching posterior head margin (SI 69–74). Eyes relatively large (OI 34–35). Mesosomal outline in profile relatively flat, elongate and low (LMI 35–37), moderately to strongly marginate from lateral to dorsal mesosoma; promesonotal suture absent; metanotal groove present, distinct, but relatively shallow. Propodeum armed with short, triangular to elongate-triangular, and acute teeth (PSLI 16–18), propodeal lobes reduced, short, and well rounded, usually shorter than propodeal teeth. Tibiae and femorae strongly swollen. Petiolar node nodiform with moderately rounded antero- and posterodorsal margins, in profile around 1.1 times higher than long (LPeI 88–93), anterior and posterior faces approximately parallel, anterodorsal and posterodorsal margins situated at about same height and equally angled, petiolar dorsum clearly convex; node in dorsal view around 1.2 to 1.3 times longer than wide (DPeI 77–81), in dorsal view pronotum between 2.0 and 2.1 times wider than petiolar node (PeNI 48–49). Postpetiole in profile globular, approximately 1.2 to 1.3 times higher than long (LPpI 75–86); in dorsal view between 1.2 and 1.3 times wider than long (DPpI 122–125), pronotum around 1.4 times wider than postpetiole (PpNI 69–70). Postpetiole in profile more or less of same volume as petiolar node, postpetiole in dorsal view around 1.4 times wider than petiolar node (PPI 141–145). Mandibles and clypeus unsculptured, smooth, and shining; cephalic dorsum between frontal carinae with fine irregularly longitudinally rugulose/rugose sculpture, rugulae/rugae often interrupted, meandering, or with cross-meshes, cephalic dorsum also puncticulate to punctate throughout its length; scrobal area strongly reticulate-punctate; lateral head mainly reticulate-rugose with weak to moderately well developed punctate ground sculpture. Ground sculpture on head usually weak, except scrobal area (see above). Dorsum of mesosoma densely longitudinally rugose on top of strong punctate ground sculpture; lateral mesosoma longitudinally rugose and very conspicuously reticulate-punctate. Forecoxae unsculptured, smooth, and shining. Petiolar node and postpetiole superficially longitudinally rugulose or irregularly rugulose superimposed on conspicuous but relatively weak reticulate-punctate ground sculpture. Mesosoma and waist segments appearing matt. First gastral tergite unsculptured, smooth, and shiny. Pilosity and pubescence greatly reduced: head with few pairs of moderately long, standing hairs, anterior pronotum with one long pair, waist segments sometimes with one long pair each, and sometimes first gastral tergite with one long pair; appressed pubescence present everywhere on body, but noticeable only on antennae, cephalic dorsum, legs, and first gastral tergite. Anterior edges of antennal scapes and dorsal (outer) surfaces of hind tibiae with appressed hairs. Body strongly bicoloured with dark brown to black gaster contrasting with light brown to reddish brown remainder.

#### Distribution and biology.

So far, *Tetramorium uelense* is known from a few collections in savannah habitats throughout a relatively wide geographical range from West to Central Africa (Fig. 3). The known distribution spans Guinea through Ghana and Nigeria to the northeast of the D. R. Congo close to South Sudan and Uganda. Compared to most other Afrotropical *Tetramorium* species, there is a wealth of information about the natural history of *Tetramorium uelense* (Longhurst, 1979). Longhurst et al. (1979) provided important observation data about nests, foraging, recruitment, and predation on termites. *Tetramorium uelense* live in subterranean nests difficult to locate without observing foraging workers. At least in the area observed by Longurst et al. (1979), the main prey of *Tetramorium uelense* consisted of various species of *Microtermes* Wasmann, and *Tetramorium uelense* exerted great predation pressure on these small termites. Scouting is performed by solitary workers that search the leaf litter, fallen grass stems or pieces of wood for prey. After locating termites the scouts return to the colony for recruitment of groups between 10 to 30 workers. These groups locate, immobilise, and retrieve the prey. For more details refer to Longhurst et al. (1979).

#### Discussion.

*Tetramorium uelense* can be easily distinguished from the remainder of the *Tetramorium decem* species group. The presence of longitudinally rugose sculpture on the dorsum of the mesosoma separates *Tetramorium uelense* immediately from *Tetramorium decem*, *Tetramorium ultor*, and *Tetramorium venator*. In the latter three the mesosomal dorsum is completely unsculptured, smooth, and very shiny. The only other species with sculpture on the dorsum of the mesosoma, which could be confused with *Tetramorium uelense*, is *Tetramorium raptor*. Nevertheless, both are well separable in morphology and ecology. Most obviously, *Tetramorium uelense* is a larger species (WL 0.98–1.06) with distinct bicolouration while *Tetramorium raptor* (WL 0.88–0.93) is smaller and a uniform dark brown colour. In addition, *Tetramorium uelense* has longer and better developed propodeal teeth (PSLI 16–18) compared to *Tetramorium raptor* (PSLI 10–11), even though this might be difficult to see and may require measurements to confirm. Another, more visible character is the sculpture on the mesosomal dorsum, which is strongly longitudinally rugose with distinct punctate ground sculpture in *Tetramorium uelense* versus longitudinally rugulose with very little ground sculpture in *Tetramorium raptor*. Also, the lateral pronotum of the latter is mostly unsculptured, smooth, and shiny while in *Tetramorium uelense* the lateral pronotum is strongly rugose with conspicuous ground sculpture.

#### Variation.

Despite the broad distribution range, we did not observe any significant intraspecific variation in *Tetramorium uelense*.

### Tetramorium ultor

Forel, 1913b comb. r., stat. r. & stat. n.

**Figure 11.**
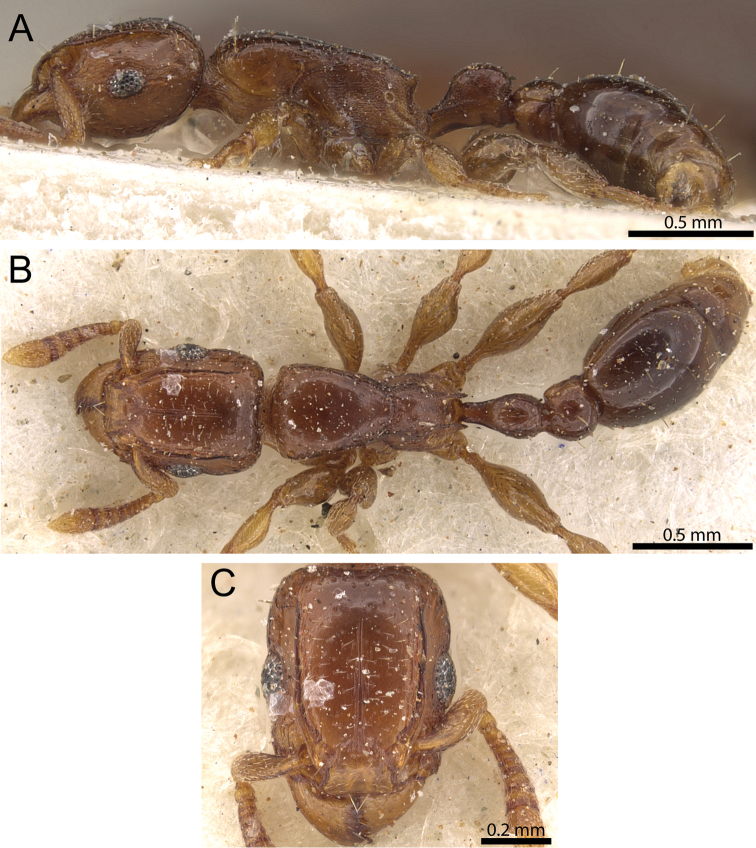
*Tetramorium ultor* paralectotype worker (CASENT0901036). **A** Body in profile **B** Body in dorsal view **C** Head in full-face view.

Figs 3 , 4C , 7A , 7B , 11

Tetramorium (Decamorium) decem ultor Forel, 1913b: 217. [Combination in *Decamorium* by Wheeler 1922: 906; junior synonym of *Decamorium decem* by Bolton 1976: 298.]

#### Type material.

**Lectotype** [designated here], pinned worker, ZIMBABWE, Shiloh, 19.73333S, 28.55E, 12.V.1913 (*G. Arnold*) (MHNG: CASENT0909197) [examined]. **Paralectotypes**, seven pinned workers with same data as lectotype (BMNH: CASENT0901036; MHNG: CASENT0195688) [examined].

[Note: the GPS data of the type locality was not provided by the locality label or the original description. The data presented above is based on our own geo-referencing of the Shiloh locality located in Matabeleland North province. Consequently, it should be considered an approximation and not the exact position of the type locality.]

#### Non-type material.

MOZAMBIQUE: Sofala Province, Gorongosa National Park, Limestone Gorge, 18°57'13"S, 34°10'37.6"E, 81 m, 15.V.2012 (*G.D. Alpert & E.O. Wilson*); Sofala Province, Gorongosa National Park, 5 km S Chitango, 18°59'28.8"S, 34°21'10"E, 10 m, secondary forest, 1.VI.2012 (*G.D. Alpert*); Sofala Province, Gorongosa National Park, Centracao Outpost (Piva-Joao), 18°30'20"S, 34°29'7"E, small forest along river, 11.VI.2012 (*D. Muala & T. Torcida*); Sofala Province, Gorongosa National Park, WP092, 18°56.1'3.1"S, 34°23'36.7"E, 51 m, open area, 26.VI.2012 (*G.D. Alpert*); KENYA: Kwale District, Shimba Hills, Longomagandi National Reserve, 4.23S, 39.43E, primary hardwood forest, 2.VI.2001 (*R.R. Snelling*); TANZANIA: Pwani, Rufiji District, Kichi Hills Forest Reserve, 8.23889S, 38.65023E, 499 m, primary forest, 5.–7.III.2008 (*P. Hawkes, Y. Mlacha, & F. Ninga*); ZAMBIA: Southern Province, 16.79533S, 26.93833E, 1330 m, Choma, Gwembe Lodge, miombo woodland, 3.XII.2005 (*B.L. Fisher*); ZIMBABWE: Balla-Balla, 20.45S, 29.03E, 1.IV.1945; Umtali, II.1917 (*G. Arnold*).

#### Diagnosis.

*Tetramorium ultor* can be recognised by the following combination of characters: relatively smaller species (WL 0.85–0.96); very large eyes (OI 33–36); propodeum armed with short teeth (PSLI 10–13); petiolar node in profile around 1.1 to 1.2 times higher than long (LPeI 86–92); dorsum of promesonotum unsculptured, smooth, and very shiny; body of uniform light to chestnut brown, appendages often lighter.

#### Worker measurements

**(N=25).** HL 0.62–0.70 (0.66); HW 0.48–0.58 (0.53); SL 0.35–0.42 (0.37); EL 0.16–0.20 (0.19); PH 0.29–0.33 (0.30); PW 0.37–0.45 (0.41); WL 0.85–0.96 (0.89); PSL 0.07–0.09 (0.08); PTL 0.22–0.25 (0.24); PTH 0.25–0.29 (0.27); PTW 0.19–0.22 (0.20); PPL 0.19–0.23 (0.21); PPH 0.25–0.30 (0.27); PPW 0.24–0.30 (0.27); CI 77–82 (80); SI 67–73 (70); OI 33–36 (35); DMI 44–48 (46); LMI 32–35 (34); PSLI 10–13 (12); PeNI 46–50 (48); LPeI 86–92 (88); DPeI 79–86 (84); PpNI 60–71 (67); LPpI 73–81 (78); DPpI 126–132 (130); PPI 130–145 (139).

#### Worker description.

Head much longer than wide (CI 77–82); posterior head margin weakly concave. Anterior clypeal margin with distinct, but often shallow median impression. Frontal carinae strongly developed and noticeably raised forming dorsal margin of very well-developed antennal scrobes, curving down ventrally and anteriorly halfway between posterior eye margin and posterior head margin and forming posterior and ventral scrobe margins; antennal scrobes very well developed, deep and with clearly defined margins all around, median scrobal carina absent. Antennal scapes short, not reaching posterior head margin (SI 67–73). Eyes very large (OI 33–36). Mesosomal outline in profile relatively flat, long and low (LMI 32–35), moderately marginate from lateral to dorsal mesosoma; promesonotal suture absent; metanotal groove present and distinct, but relatively shallow. Propodeum armed with short, triangular, and mostly blunt teeth (PSLI 10–13), propodeal lobes short, triangular, and usually blunt, in profile usually longer and more voluminous than propodeal spines. Tibiae and femorae strongly swollen. Petiolar node nodiform with moderately rounded antero- and posterodorsal margins, in profile around 1.1 to 1.2 times higher than long (LPeI 86–92), anterior and posterior faces approximately parallel, anterodorsal and posterodorsal margins situated at about same height and equally angled, petiolar dorsum weakly convex; node in dorsal view around 1.1 to 1.2 times longer than wide (DPeI 79–86), in dorsal view pronotum between 2.0 to 2.2 times wider than petiolar node (PeNI 46–50). Postpetiole in profile globular, approximately 1.2 to 1.4 times higher than long (LPpI 73–81); in dorsal view around 1.3 times wider than long (DPpI 126–132), pronotum approximately 1.4 to 1.5 times wider than postpetiole (PpNI 60–71). Postpetiole in profile appearing less voluminous than petiolar node, postpetiole in dorsal view between 1.3 to 1.5 times wider than petiolar node (PPI 130–145). Mandibles and clypeus usually fully unsculptured, smooth, and shining; cephalic dorsum between frontal carinae mostly unsculptured and shiny, median ruga present and distinct, cephalic dorsum also puncticulate to punctate throughout its length, close to posterior head margin especially pronounced; scrobal area unsculptured, smooth, and very shiny; lateral head ventral of antennal scrobe mainly reticulate-rugose; ground sculpture on head usually weak to absent. Dorsum of mesosoma mostly unsculptured, smooth, and shiny with scattered punctures, rarely with few traces of rugulae; lateral mesosoma mostly unsculptured and shiny, posteriorly irregularly rugose and conspicuously reticulate-punctate. Petiolar node and postpetiole only weakly sculptured, laterally usually superficially rugulose and punctate on lower half and more unsculptured on upper half, node dorsally mostly smooth; postpetiole mostly unsculptured, smooth, and shiny with scattered punctures. First gastral tergite unsculptured, smooth, and shiny. Pilosity and pubescence greatly reduced: head with few pairs of moderately long, standing hairs, anterior pronotum with one long pair, waist segments sometimes with one long pair each, and sometimes first gastral tergite with one long pair; appressed pubescence present everywhere on body, but noticeable only on antennae, cephalic dorsum, legs, and first gastral tergite. Anterior edges of antennal scapes and dorsal (outer) surfaces of hind tibiae with appressed hairs. Body uniformly brown, appendages often lighter.

#### Distribution and biology.

*Tetramorium ultor* is widespread in eastern and southern Africa (Fig. 3). It is distributed from Kenya south to Mozambique, and also found in Zambia and Zimbabwe. Most localities are tropical dry forest habitats or miombo woodland. Also, *Tetramorium ultor* seems to be a ground-active species nesting in or under rotten logs and is likely termitophagous like the other group members.

#### Discussion.

Since Bolton (1976) synonymised *Tetramorium ultor* under *Tetramorium decem*, almost all of the material of *Tetramorium ultor* examined here was identified and/or labelled as *Tetramorium decem* prior to this study. However, after careful examination of all the available material, we have come to the conclusion that *Tetramorium ultor* is distinctive enough to merit species status. *Tetramorium ultor* is smaller (WL 0.85–0.96), has shorter propodeal teeth (PSLI 10–13), a lower petiolar node, around 1.1 to 1.2 times higher than long (LPeI 86–92), and is of uniform light brown to chestnut brown body colouration. By contrast, *Tetramorium decem* is larger (WL 1.02–1.16), has longer propodeal spines (PSLI 17–19), a higher petiolar node, in profile around 1.2 to 1.3 times higher than long (LPeI 77–82), and is conspicuously bicoloured. In addition, both species share most of their distribution range without any intermediate forms. Furthermore, *Tetramorium ultor* is unlikely to be confused with *Tetramorium raptor* and *Tetramorium uelense* since the latter two have a conspicuously rugose/rugulose promesonotum, which is completely unsculptured, smooth and shiny in *Tetramorium ultor*. The last species of the group, *Tetramorium venator*, is the one most similar to *Tetramorium ultor*, and both species are allopatric. However, both species can be separated by eye size, colour, and a different habitat choice. *Tetramorium venator* has larger eyes (OI 37–40) and is of a much darker brown than *Tetramorium ultor*, which has smaller eyes (OI 33–36) and is of a lighter brown. In addition, the latter species is more arid-adapted, occurring in woodlands and dry forests while *Tetramorium venator* seems to be a forest specialist found in primary, secondary, or disturbed rainforests. We consider the above arguments as sufficient to justify the heterospecificity of both species. Further arguments are provided below in the description of *Tetramorium venator*.

#### Variation.

Based on the available material, we did not observe any intraspecific variation in *Tetramorium ultor*.

### Tetramorium venator

Hita Garcia sp. n.

http://zoobank.org/02C5E77F-FFD9-4204-843C-1E541B84972A

http://species-id.net/wiki/Tetramorium_venator

**Figure 12.**
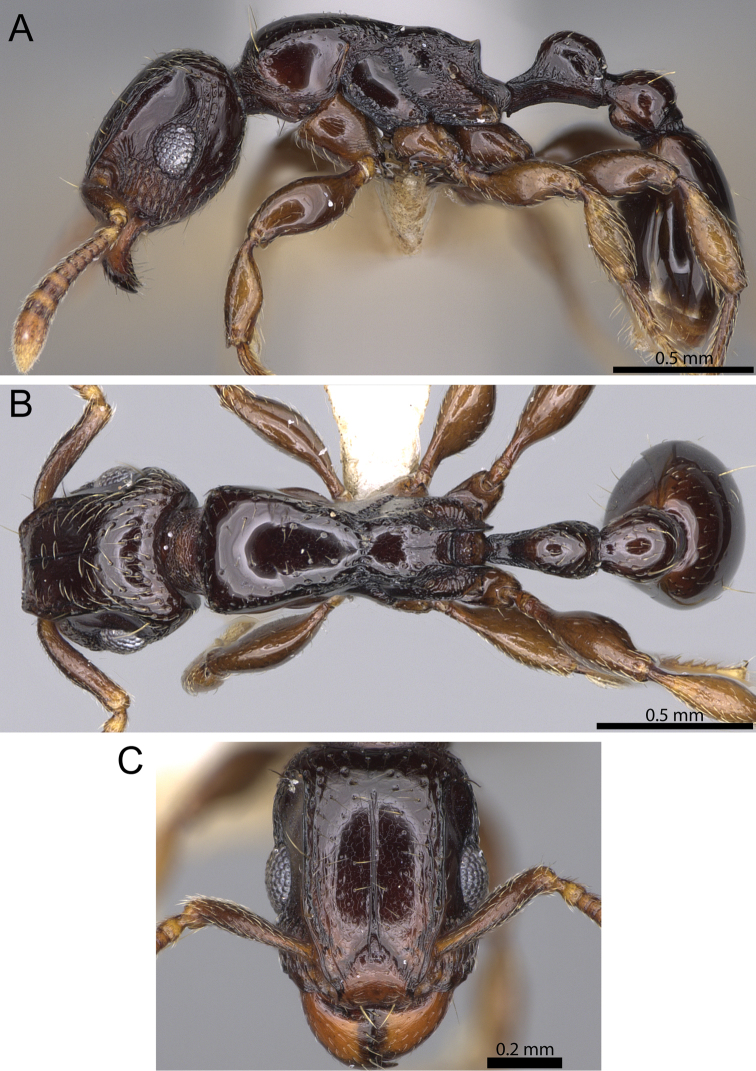
*Tetramorium venator* holotype worker (CASENT0195574). **A** Body in profile **B** Body in dorsal view **C** Head in full-face view.

Figs 3 , 4D , 6B , 7C , 7D , 12

#### Type material.

Holotype, pinned worker, KENYA, Western Kenya, Kakamega Forest, Bunyala Forest Fragment, 0.37889N, 34.69917E, 1448 m, disturbed primary forest, Kakamega 2008 survey, leaf litter, pitfall trap, Transect 35, position 10 m, 1.VIII.2008 (*G. Fischer*) (CASC: CASENT0195574). Paratypes, six pinned workers with same data as holotype (BMNH: CASENT0195625; CASC: CASENT0217165; BMNH: CASENT0195625; LACM: CASENT0195627; MCZ: CASENT0195624; NMK: CASENT0195626; ZFMK: CASENT0195623).

#### Non-type material.

CAMEROON: Centre, Mbalmayo, 1.XI.1993 (*N. Stork*); Centre, Ottotomo, 24.IV.1986 (*A. Dejean*); Est, Abong Mbang, 28.VI.1988 (*A. Dejean*); Sud, Bondé Forest, N’kolo village, 27.5 km 155°SSE Elogbatindi, 3.22167N, 10.24667E, 40 m, rainforest, 12.IV.2000 (*B.L. Fisher*); Sud, Res. de Faune de Campo, Massif des Mamelles, 15.1 km 84°E Ébodjé, 2.59417N, 9.9595E, 180 m, rainforest, 4.IV.2000 (*B.L. Fisher*); Sud, Res. de Faune de Campo, 2.16 km 106°ESE Ébodjé, 2.56783N, 9.84433E, 10 m, littoral rainforest, 9.IV.2000 (*B.L. Fisher*); Sud, P. N. Campo, 43.3 km 108°ESE Campo, 2.2825N, 10.20617E, 290 m, rainforest, 7.IV.2000 (*B.L. Fisher*); Sud-Ouest, Bimbia Forest, 7.4 km 119°ESE Limbe, 3.98183N, 9.2625E, 40 m, 14.IV.2000 (*B.L. Fisher*); Sud-Ouest, Korup N. P., 6.9 km 317°NW Mundemba, 5.016N, 8.864E, 110 m, rainforest, 19.IV.2000 (*B.L. Fisher*); CENTRAL AFRICAN REPUBLIC: Prefecture Sangha-Mbaéré, Parc National Dzanga-Ndoki, Mabéa Bai, 21.4 km 53°NE Bayanga, 3.03333N, 16.41E, 510 m, rainforest, 1.–7.V.2001 (*B.L. Fisher*); DEMOCRATIC REPUBLIC OF CONGO: Epulu, 750 m, 1.38333N, 28.58333E, rainforest, 1.XI.1995 (*S.D. Torti*); Kikwit, Kinzambi, 27.III.1984 (*A. Dejean*); 44 miles E. of Kileba, 1110 m, 16.I.1958 (*E.S. Ross & R.E. Leech*); GABON: La Makandé Forêt des Abeilles, 1.I.–1.II.1999 (*S. Lewis*); Ogooué-Ivindo, Makokou, C.N.R.S., 10.VII.1974 (*W. Gotwald*); Ogooue-Maritime, Réserve des Monts Doudou; 24.3km 307°NW Doussala, 2.2225S, 10.40583E, 370 m, coastal lowland rainforest, 5.–12.III.2000 (*S. van Noort*); Ogooue-Maritime, Aire d’Exploit. Rationnelle de Faune des Monts Doudou, 24.3 km 307°NW Doussala, 2.22639S, 10.40972E, 375 m, rainforest, 6.III.2000 (*B.L. Fisher*); Ogooue-Maritime, Reserve de la Moukalaba-Dougoua, 12.2 km 305°NW Doussala, 2.28333S, 10.49717E, 110 m, coastal lowland rainforest, sited within forest, 24.II.-3.III.2000 (*S. van Noort*); Ogooue-Maritime, Reserve de Faune de la Moukalaba-Dougoua, 10.8 km 214°SW Doussala, 2.42267S, 10.54533E, 110 m, rainforest, 29.II.2000 (*B.L. Fisher*); Ogooue-Maritime, Reserve de Faune de la Moukalaba-Dougoua, 12.2 km 305°NW Doussala, 2.31667N, 10.53333E, 110 m, rainforest, 1.III.2000 (*B.L. Fisher*); Woleu-Ntem, 31.3 km 108°ESE Minvoul, 2.08N, 12.40667E, 600 m, rainforest, 7.–15.II.1998 (*B.L. Fisher*); GHANA: Akwapim, Tafo, 19.I.1970 (*B. Bolton*); Ashanti, Poano, cocoa, 9.IX.1992 (*R. Belshaw*); Atewa Forest Reserve, near Kibi, primary forest, 24.III.1992 (*R. Belshaw*); Eastern, Kade, 1.I.1992 (*R. Belshaw*); Enchi, 17.V.1969 (*D. Leston*); Esunkawkaw Forest Reserve, primary forest, 27.X.1992 (*R. Belshaw*); Nkawanda near Nkawkaw, secondary forest, 12.XII.1991 (*R. Belshaw*); Portrase, 1.III.1992 (*R. Belshaw*); KENYA: Western Kenya, Kakamega Forest, Bunyala Forest Fragment, 0.37889N, 34.69917E, 1448 m, disturbed primary forest, 1.VIII.2008 (*G. Fischer*); LIBERIA: Monrovia, 5.VII.1957 (*E.S. Ross & R.E. Leech*); TANZANIA: Kigoma Region, Gombe Stream National Park, 4.7S, 29.616667E, 915–1012 m, 29.XII.2009–12.I.2010 (*R. O’Malley*); UGANDA: Semuliki NP, 00.83556, 30.15542 ± 200 m, 676 m, rainforest, 30.–31.VII.2012 (*B.L. Fisher et al.*); Semuliki NP, 00.84483, 30.15052 ± 200 m, 680 m, rainforest, 2.VIII.2012 (*B.L. Fisher et al.*).

#### Diagnosis.

*Tetramorium venator* can be recognised by the following combination of characters: relatively smaller species (WL 0.87–0.98); very large eyes, largest in the group (OI 37–40); propodeum armed with very short, triangular, and moderately acute (PSLI 9–12); petiolar node in profile between 1.0 to 1.2 times higher than long (LPeI 90–100); dorsum of promesonotum unsculptured, smooth, and very shiny; head, mesosoma, waist segments, and gaster uniformly very dark brown to black, appendages of lighter brown.

#### Worker measurements

(N=25). HL 0.64–0.71 (0.67); HW 0.51–0.59 (0.54); SL 0.38–0.43 (0.40); EL 0.19–0.22 (0.21); PH 0.30–0.36 (0.32); PW 0.39–0.45 (0.41); WL 0.87–0.98 (0.92); PSL 0.06–0.09 (0.07); PTL 0.23–0.26 (0.25); PTH 0.25–0.29 (0.26); PTW 0.18–0.22 (0.20); PPL 0.21–0.25 (0.22); PPH 0.25–0.30 (0.26); PPW 0.25–0.30 (0.26); CI 79–83 (81); SI 70–75 (74); OI 37–40 (38); DMI 43–46 (44); LMI 33–37 (35); PSLI 9–12 (10); PeNI 45–51 (48); LPeI 90–100 (93); DPeI 76–85 (80); PpNI 63–67 (65); LPpI 80–86 (84); DPpI 115–124 (119); PPI 130–144 (135).

#### Worker description.

Head much longer than wide (CI 79–83); posterior head margin weakly concave. Anterior clypeal margin with distinct, but often shallow median impression. Frontal carinae strongly developed and noticeably raised forming dorsal margin of very well-developed antennal scrobes, curving down ventrally and anteriorly halfway between posterior eye margin and posterior head margin and forming posterior and ventral scrobe margins; antennal scrobes very well developed, deep and with clearly defined margins all around, median scrobal carina absent. Antennal scapes short, not reaching posterior head margin (SI 70–75). Eyes very large (37–40). Mesosomal outline in profile relatively flat, long and low (LMI 33–37), moderately marginate from lateral to dorsal mesosoma; promesonotal suture absent; metanotal groove present and distinct, but relatively shallow. Propodeum armed with very short, triangular, and moderately acute teeth (PSLI 9–12), propodeal lobes short, triangular to rounded, and usually blunt, in profile more or less of same length as propodeal teeth and appearing more voluminous than propodeal spines. Tibiae and femorae strongly swollen. Petiolar node nodiform with moderately rounded antero- and posterodorsal margins, in profile between 1.0 to 1.2 times higher than long (LPeI 90–100), anterior and posterior faces approximately parallel, anterodorsal and posterodorsal margins situated at about same height and equally angled, petiolar dorsum usually conspicuously convex, sometimes only weakly so; node in dorsal view around 1.2 to 1.3 times longer than wide (DPeI 76–85), in dorsal view pronotum between 2.0 to 2.2 times wider than petiolar node (PeNI 45–51). Postpetiole in profile globular, approximately 1.2 times higher than long (LPpI 80–86); in dorsal view around 1.2 times wider than long (DPpI 115–124), pronotum approximately 1.5 to 1.6 times wider than postpetiole (PpNI 63–67). Postpetiole in profile appearing less voluminous than petiolar node, postpetiole in dorsal view between 1.3 to 1.5 times wider than petiolar node (PPI 130–144). Mandibles and clypeus usually fully unsculptured, smooth, and shining; cephalic dorsum between frontal carinae mostly unsculptured and shiny, median ruga present and distinct, cephalic dorsum also puncticulate to punctate across its length, close to posterior head margin especially pronounced; scrobal area unsculptured, smooth and very shiny; lateral head ventral of antennal scrobe mainly reticulate-rugose; ground sculpture on head usually weak to absent. Dorsum of mesosoma mostly unsculptured, smooth and shiny with scattered punctures, rarely with few traces of rugulae; lateral mesosoma mostly unsculptured and shiny, posteriorly irregularly rugose and conspicuously reticulate-punctate. Petiolar node and postpetiole only weakly sculptured, laterally usually superficially rugulose and punctate on lower half and more unsculptured on upper half, node dorsally mostly smooth; postpetiole mostly unsculptured, smooth and shiny with scattered punctures. First gastral tergite unsculptured, smooth, and shiny. Pilosity and pubescence greatly reduced: head with few pairs of moderately long, standing hairs, anterior pronotum with one long pair, waist segments sometimes with one long pair each, and sometimes first gastral tergite with one long pair; appressed pubescence present everywhere on body, but noticeable only on antennae, cephalic dorsum, legs, and first gastral tergite. Anterior edges of antennal scapes and dorsal (outer) surfaces of hind tibiae with appressed hairs. Head, mesosoma, waist segments, and gaster uniformly very dark brown to black, appendages of lighter brown.

#### Etymology.

The name of the new species is Latin and means “hunter” referring to the predatory lifestyle of *Tetramorium venator*. The species epithet is a nominative noun, and thus invariant.

#### Distribution and biology.

*Tetramorium venator* is the most widespread and abundant species of the group. It is found throughout much of the equatorial forest belt from Liberia in the west to Kenya in the east (Fig. 3). Even though there was no material from Benin, Togo, Nigeria, Equatorial Guinea or South Sudan available for this study, we expect that *Tetramorium venator* will be found in most or all of these countries. Based on the available data, this species lives in the leaf litter stratum of primary, secondary, or disturbed rainforests. Additionally, *Tetramorium venator* seems to be found at lower elevations in West and Central Africa, but also occurs at mid elevations further east in the eastern D.R. Congo, Tanzania, and Kenya, where it reaches its highest known elevation at the type locality at 1448 m. Based on unpublished stable isotope data from the type series, *Tetramorium venator* is a predatory species, and we assume that it feeds on termites. This is supported by some series from Cameroon that were collected while foraging in the nests of *Cubitermes* Wasmann.

#### Discussion.

Despite being common and collected fairly often prior to this study, most of the material of *Tetramorium venator* was identified and labelled as *Tetramorium decem*. Indeed, more than 90% of all the material listed as the latter species at the beginning of our revision turned out to be *Tetramorium venator*. Nevertheless, our revision shows that they are clearly not conspecific. *Tetramorium venator* is smaller in size (WL 0.87–0.98), has larger eyes (OI 37–40), shorter propodeal teeth (PSLI 9–12), a lower petiolar node (LPeI 90–100), and has a uniform body colouration. By contrast, *Tetramorium decem* is larger (WL 1.02–1.16), has smaller eyes (OI 32–34), longer propodeal spines (PSLI 17–19), a higher petiolar node (LPeI 77–82), and is distinctly bicoloured. Also, *Tetramorium venator* is a rainforest species while *Tetramorium decem* lives in savannah or woodland.

The abovementioned very large eyes of *Tetramorium venator* separate it also from *Tetramorium ultor*, which has smaller eyes (OI 33–36). In addition, *Tetramorium ultor* is also of a much lighter colour, usually light brown to chestnut brown, and prefers dry forest or woodland habitats. It should be noted, however, that *Tetramorium ultor* and *Tetramorium venator* are morphologically very close to each other and differ significantly only in eye size, colour and habitat preference. They could represent different ecotypes of the same species, one adapted to more shaded and humid forest versus one specialised to more arid savannah, woodland, and dry forest. Nevertheless, if this was true, then we would see some intermediate forms in transitional habitats, and there are none at present. As a matter of fact, *Tetramorium venator* is also found in secondary and disturbed rainforests. The type series was collected in a highly disturbed rainforest fragment in Kenya and the material from Gombe in Tanzania is from a rainforest-woodland mosaic. Both species are also separated by the Great Rift Valley, which separates different faunistic sub-regions of the Afrotropical region. We consider *Tetramorium venator* as a faunal element of the Guineo-Congolian forest zone, while we believe *Tetramorium ultor* is a species of the arid corridor running from East to Southern Africa. Based on the available material and African biogeography in general, we conclude that our two species hypothesis is more likely.

Furthermore, *Tetramorium venator* cannot be misidentified with either *Tetramorium uelense* or *Tetramorium raptor* since both possess strongly developed rugulose/rugose sculpture on the promesonotal dorsum that is absent in *Tetramorium venator*. At present, *Tetramorium venator* overlaps in its distribution with *Tetramorium uelense* and *Tetramorium raptor* in West and Central Africa. We think it might also overlap with *Tetramorium decem* and *Tetramorium ultor* in East Africa, even though it currently seems as if they are widely separated geographically. However, since the sampling is very patchy, especially in East Africa, much more *Tetramorium decem* and *Tetramorium ultor* material is likely to be collected with further inventories, and these two species will be found in close proximity to *Tetramorium venator*. Nevertheless, the latter species is restricted to more humid forest habitats, whereas *Tetramorium decem* and *Tetramorium ultor* clearly prefer more arid savannah, grassland, woodland and tropical dry forest.

#### Variation.

Intriguingly, even though *Tetramorium venator* is very broadly distributed in Equatorial Africa, there seems to be no significant intraspecific variation.

## Supplementary Material

XML Treatment for Tetramorium decem

XML Treatment for Tetramorium raptor

XML Treatment for Tetramorium uelense

XML Treatment for Tetramorium ultor

XML Treatment for Tetramorium venator
